# Partial amputation regrowth of P4 distal digit in an eastern grey kangaroo (*Macropus giganteus*): a case report

**DOI:** 10.1111/avj.70108

**Published:** 2026-06-25

**Authors:** L Cummins, G Cummins, J Clough, A Shen

**Affiliations:** ^1^ Faculty of Science, Medicine and Health University of Wollongong Wollongong New South Wales Australia; ^2^ Possumwood Wildlife Hospital Superintendant Veterinarian Bungendore New South Wales Australia

## Abstract

This case report describes distal regrowth of the fourth hind digit (P4) following traumatic autoamputation in a young adult eastern grey kangaroo (*Macropus giganteus*). Injury to P4 is generally considered to carry a poor prognosis due to its critical role in weight‐bearing and propulsion during hopping. Following loss of the distal phalanx, the nail bed remained intact and healing progressed without surgical intervention. Sequential observations documented epithelialisation, preservation of the nail bed and subsequent formation of a keratinised nail structure. The animal maintained normal locomotion and body condition throughout recovery, including successfully carrying a pouched young. Long‐term follow‐up confirmed survival and reproduction more than 2 years post‐release. While histological confirmation was not available, the observed pattern of tissue replacement is suggestive of a regenerative‐like response rather than conventional fracture repair. This case highlights the importance of functional assessment over anatomical completeness in rehabilitation decision‐making and suggests potential under‐recognised plasticity in macropod tissue healing.

**Abbreviations**
P4: fourth hind digit
EGK: Eastern Grey Kangarooe

The fourth hind digit (P4; digit IV), together with its associated metatarsal, forms the principal weight‐bearing and propulsive element of the hind foot in eastern grey kangaroos (*Macropus giganteus*). Macropod hind limb morphology is highly specialised for bipedal hopping, with the long axis of the limb aligned through P4 to provide an efficient mechanical pathway for the transmission of forces generated during locomotion, particularly at speed.^1^ As a consequence, injuries affecting the P4 digit or distal metatarsal are widely regarded as carrying a poor prognosis for successful rehabilitation, as impairment of this structure may compromise hopping efficiency and survival in the wild.

In macropods, as in other mammals, skeletal injury typically heals through conventional reparative processes involving callus formation and bone remodelling, resulting in restoration of structural integrity rather than replacement of lost anatomy.^2^ In contrast, spontaneous regeneration of amputated appendicular structures is extremely limited in mammals. While extensive limb regeneration is characteristic of some amphibians, most mammals demonstrate little regenerative capacity beyond routine fracture repair. Rare exceptions have been documented in rodent models and in paediatric human and non‐human primate cases, where partial regrowth of distal digit tips may occur when the nail bed remains intact.^3^, ^4^, ^5^, ^6^ To our knowledge, there are no published reports describing distal digit or metatarsal regrowth in marsupials.

From a wildlife management perspective, injuries to the hind limb, particularly those affecting principal weight‐bearing structures, raise important ethical and policy considerations. Australian wildlife rehabilitation guidelines consistently emphasise that macropods should only be released if they demonstrate normal locomotion, effective foraging and absence of ongoing pain or disability.^7^ Although these guidelines do not explicitly reference individual digits, injuries to P4 that result in persistent lameness, altered gait or compromised weight‐bearing would generally be considered incompatible with release at initial assessment. Importantly, however, these frameworks prioritise functional outcome rather than anatomical completeness when determining suitability for release.

This case provides rare insight into macropod limb healing and may inform future research into the extent of tissue plasticity in marsupials, the potential role of the nail organ in directing distal tissue regrowth, and the implications for clinical decision‐making and rehabilitation outcomes following distal limb injury. The findings are presented to inform comparative mammalian biology while remaining consistent with existing wildlife rehabilitation principles.

## Clinical features

**Figure 1:**
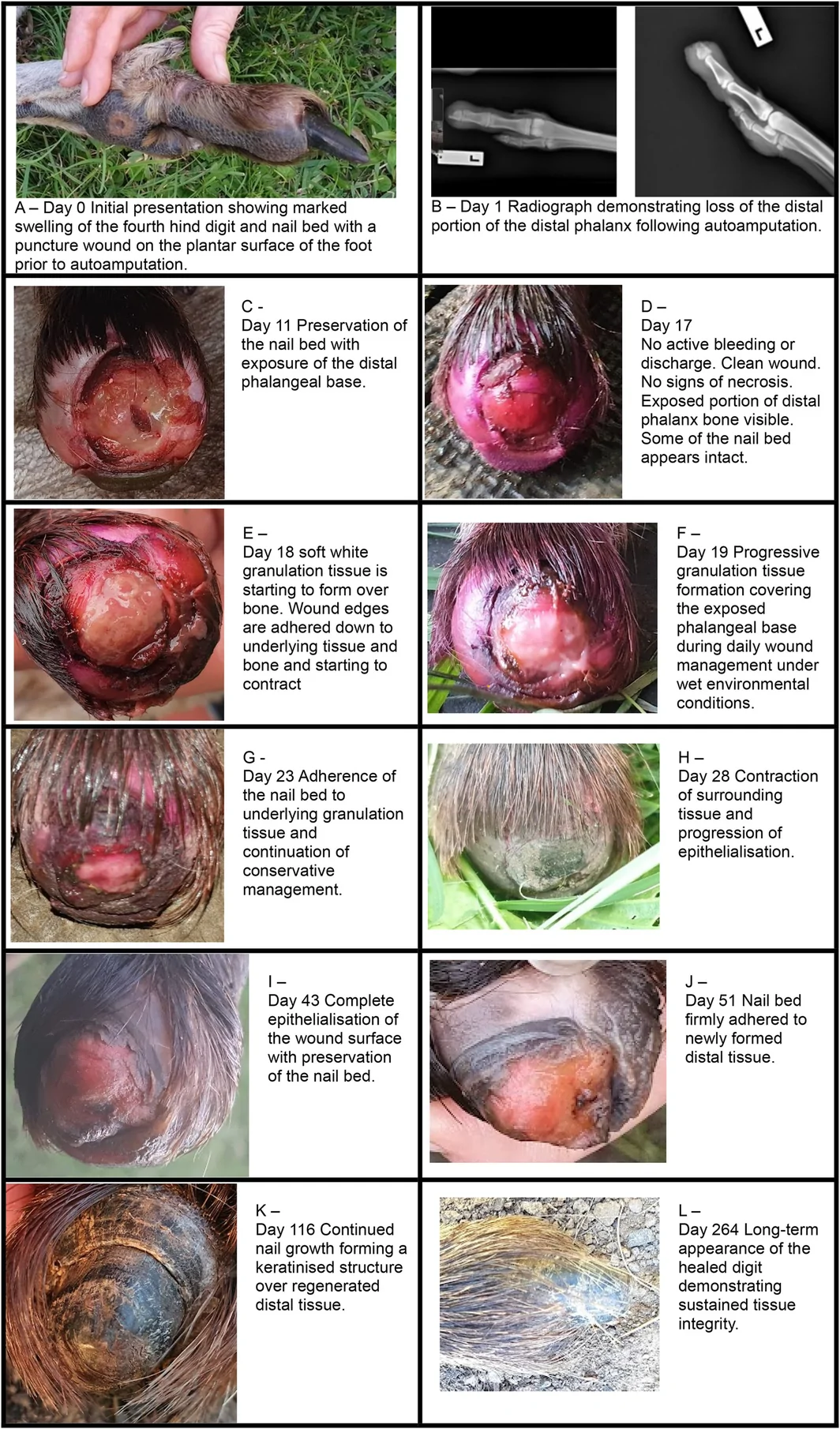
Sequential photographic and radiographic images demonstrating healing and distal regrowth of the fourth hind digit (P4) following traumatic autoamputation in a young adult female eastern grey kangaroo (EGK). Pink staining visible in some images represents topical application of Cetrigen spray used during wound management.

An approximately 18‐kg, 2‐year‐old female eastern grey kangaroo (*Macropus giganteus*) was observed in February 2022 following trauma to the plantar surface of the hind foot (Figure 1A, Day 0). The animal had been hand‐reared from an estimated body mass of 1.5 kg and released onto a private property in regional New South Wales approximately 1 month prior to injury.

At initial observation, the toenail of the fourth hind digit (P4) was intact. Marked swelling of the surrounding tissue was present, with a visible puncture wound on the plantar pad consistent with trauma from a sharp object such as barbed wire. The lesions were not actively bleeding and appeared consistent with an injury sustained several days prior. The kangaroo was observed intermittently chewing at the affected digit; however, no lameness or impairment of mobility was evident. Following consultation with a local veterinarian, the animal was monitored within a large, fenced enclosure containing conspecifics from her mob.

One day later, the distal portion of the toenail had autoamputated. Radiographs of the distal phalanx were obtained to assess the extent of bony involvement and to exclude osteomyelitis or significant soft tissue pathology (Figure 1B, Day 1). Throughout the treatment period, topical pink staining visible in several images represents application of Cetrigen® spray (Virbac Australia Pty Ltd, Milperra, NSW, Australia), an antibacterial and insect repellent used to minimise the risk of fly strike during open‐air wound management.

By day 11 post‐injury (Figure 1C), the nail bed remained intact and clearly visible, with exposure of the distal base of the phalanx. There was no active bleeding, purulent discharge or evidence of local infection. Between days 17 and 19 (Figure 1D–F), healing progressed favourably despite persistently wet environmental conditions that limited the ability to maintain a dry wound environment. Daily wound care was maintained, consisting of visual assessment, topical Cetrigen application and monitoring for signs of infection or tissue breakdown. Granulation tissue covered the exposed phalangeal base, with no signs of infection, tissue necrosis or wound breakdown.

By day 23 (Figure 1G), the nail bed was firmly adherent to underlying granulation tissue covering the phalangeal base. To ensure pain was monitored appropriately, the EGK was closely observed for abnormal gait, lameness, hunched posture, partially closed eyes or squinting, teeth grinding, lack of appetite and lethargy.^8^, ^9^ In the absence of infection, pain or functional impairment, a decision was made to continue conservative, open‐air management. By day 28 (Figure 1H), contraction of the surrounding skin and nail bed was evident, with epithelial tissue advancing across the granulation bed. A dry, protective crust consistent with an eschar formed over the distal wound surface between days 28 and 43 (Figure 1I). This eschar detached spontaneously as epithelialisation progressed, revealing healthy underlying tissue without evidence of infection or tissue compromise. By day 43, the wound surface was fully covered by well‐vascularised tissue, with the nail bed remaining intact. Minimal visible scarring was noted throughout the healing process.

By day 51 (Figure 1J), the nail bed was firmly adhered to newly formed tissue, with early evidence of new nail growth extending across the healed surface. Routine monitoring continued during this period, with no clinically significant changes observed until progression of nail growth became evident. By day 116 (Figure 1K), continued nail growth was evident, forming a keratinised structure over regenerated distal tissue. Long‐term follow‐up beyond day 264 (Figure 1L) demonstrated sustained integrity of the regenerated digit, with no recurrence of injury, infection or lameness.

## Treatment and management

Initial management of the distal wound was conservative. The exposed tissue was treated topically with medicinal honey, and the site was maintained in a clean, dry state where possible. Analgesia was provided using meloxicam for 3 days. Antimicrobial therapy was not administered, as there were no clinical signs of infection. The kangaroo was temporarily confined to facilitate daily wound care and close observation, including visual assessment, topical treatment and monitoring for signs of infection or tissue deterioration, while being housed in a large enclosure with access to green forage and conspecific juveniles.

Throughout the recovery period, the animal demonstrated normal ambulation and weight‐bearing, with no observable lameness. The kangaroo maintained good body condition and continued to support the growth of a pouched young, first noticed during rehabilitation. Prolonged wet environmental conditions subsequently limited the feasibility of maintaining a dry dressing.

After approximately 3 weeks, management transitioned to a conservative open‐air approach. During this phase, the distal wound was treated with Cetrigen spray to deter flies and minimise the risk of myiasis. No adverse effects were observed following this change in management.

Following adoption of open‐air management, progressive wound contraction and epithelialisation were observed. Granulation tissue remained healthy, and healing progressed without evidence of infection or tissue breakdown. By day 116 post‐injury, restoration of a keratinised nail sheath was evident, coinciding with sustained normal locomotor function.

## Discussion

This case describes an unusual instance of distal regrowth of the fourth hind digit (P4) following traumatic autoamputation in a young adult eastern grey kangaroo. To our knowledge, there are no previously published reports of comparable distal digit regrowth in macropods or other marsupials, and this observation therefore represents a novel contribution to current understanding of limb healing and tissue plasticity in this group.

The functional significance of the fourth digit in macropods makes this outcome particularly noteworthy. As the principal weight‐bearing and propulsive structure during hopping, injury to P4 is typically associated with a poor prognosis for rehabilitation and release.^7^ Consistent with this, Australian wildlife rehabilitation frameworks emphasise restoration of normal locomotion, weight‐bearing and absence of pain as key criteria for release suitability. While these guidelines do not specifically address individual digits, injuries affecting P4 that compromise gait or weight‐bearing are generally considered incompatible with successful release.

In the present case, however, functional outcomes were preserved despite initial partial loss of the distal phalanx. The animal maintained normal locomotion, demonstrated appropriate weight‐bearing and showed no behavioural indicators of compromised welfare. Long‐term follow‐up further confirmed successful reintegration into a wild population, including survival and reproduction beyond 2 years post‐release. These findings reinforce that successful rehabilitation depends on functional performance rather than complete anatomical restoration.

Healing of the P4 digit occurred without surgical intervention and was characterised by progressive epithelialisation, preservation of nail bed integrity and subsequent reformation of a keratinised nail structure. Functional recovery alone cannot be taken as evidence of biological regeneration; however, in this case, functional restoration occurred in conjunction with distal tissue replacement and renewed nail growth. The degree of tissue replacement and minimal scarring observed are more consistent with a regenerative‐like response than with conventional fracture repair, which in mammals typically proceeds via callus formation and remodelling. Similar regenerative patterns have been described in murine and human distal digit models, where preservation of the nail organ is associated with tissue regrowth rather than fibrotic repair.^5^, ^10^, ^11^

While the pattern of distal tissue regrowth observed here is suggestive of regeneration, the absence of histological or molecular analysis precludes definitive classification as true regeneration. Accordingly, the term ‘regrowth’ is used descriptively, and regeneration is discussed as a plausible interpretation rather than a confirmed mechanism. The preservation of nail bed integrity is notable, as distal digit regeneration in mammals is strongly associated with the presence of the nail organ. Experimental and mechanistic studies demonstrate that the nail epithelium and its associated stem cell populations play a key role in supporting regenerative processes, including blastema formation and bone regrowth.^10^, ^12^, ^13^ However, this relationship is not absolute; regenerative outcomes are also influenced by factors such as amputation level, local signalling environment and tissue context.^11^, ^14^ Taken together, these findings suggest that preservation of the nail organ may provide a permissive or instructive environment for distal tissue replacement, supporting a potentially conserved biological role in directing regenerative responses.

As a single observational case, these findings cannot be generalised. The animal's young age and prior hand‐rearing history may also have influenced healing capacity and should be considered when interpreting this outcome. Nevertheless, the case highlights the possibility of under‐recognised plasticity in marsupial healing responses, particularly in juvenile or young‐adult individuals.

From a clinical and rehabilitation perspective, this case does not suggest deviation from established wildlife care guidelines. Rather, it supports their emphasis on functional assessment over anatomical completeness when evaluating prognosis and release suitability. The findings indicate that not all distal injuries to the principal weight‐bearing digit should be regarded as irreversibly non‐releasable at presentation, particularly in the absence of pain, infection or functional impairment and where close monitoring is feasible before euthanasia decisions are made.

Further investigation is required to determine whether the process observed represents true regeneration or an enhanced reparative response. Documentation of comparable cases, combined with serial imaging and histological examination where possible, would help clarify the biological mechanisms involved and identify factors such as age, nutrition or mechanical loading that may influence outcomes. Evidence of regenerative‐like healing in a high‐load structure such as P4 would challenge prevailing assumptions that mammalian regeneration is restricted to low‐stress tissues and contribute to a broader understanding of tissue plasticity in marsupials.

## Conclusion

This case documents a rare instance of distal fourth digit (P4) regrowth in an eastern grey kangaroo, accompanied by restoration of normal locomotor function and long‐term survival in the wild. Preservation of nail bed integrity and absence of functional impairment were associated with progressive distal tissue replacement and reformation of a keratinised nail structure.

While limited by the absence of histological confirmation, these findings suggest a previously unreported capacity for distal tissue regrowth in macropods and support the importance of functional assessment over anatomical completeness in rehabilitation decision‐making. Further investigation is required to clarify the underlying biological mechanisms and broader relevance of this response.

## Statement of consent

Informed consent for the use of clinical data and images was obtained from the animal's carer.

## Conflict of interest and sources of funding

The authors declare no conflicts of interest or sources of funding for the work presented here.

## Data Availability

The data that support the findings of this study are available from the corresponding author upon reasonable request.
